# A review on *Q*_ST_ – *F*_ST_ comparisons of seed plants: Insights for conservation

**DOI:** 10.1002/ece3.9926

**Published:** 2023-03-28

**Authors:** Mi Yoon Chung, Juha Merilä, Yuseob Kim, Kangshan Mao, Jordi López‐Pujol, Myong Gi Chung

**Affiliations:** ^1^ Department of Biological Sciences Chungnam National University Daejeon 34134 South Korea; ^2^ Ecological Genetics Research Unit, Organismal and Evolutionary Biology Research Program, Faculty of Biological and Environmental Sciences University of Helsinki Helsinki FI‐00014 Finland; ^3^ Area of Ecology & Biodiversity School of Biological Sciences The University of Hong Kong Hong Kong SAR China; ^4^ Division of EcoScience Ewha Womans University Seoul 03760 South Korea; ^5^ Department of Life Science Ewha Womans University Seoul 03760 South Korea; ^6^ Key Laboratory for Bio‐resources and Eco‐environment of Ministry of Education, College of Life Science, State Key Laboratory of Hydraulics and Mountain River Engineering Sichuan University Chengdu 610065 China; ^7^ Botanic Institute of Barcelona (IBB), CSIC‐Ajuntament de Barcelona Barcelona 08038 Catalonia Spain; ^8^ Universidad Espíritu Santo (UEES) Samborondón 091650 Ecuador; ^9^ Division of Life Science and RINS Gyeongsang National University Jinju 52828 South Korea

**Keywords:** adaptive variation, conservation, genetic diversity, herbaceous plants, neutral variation, woody species

## Abstract

Increased access to genome‐wide data provides new opportunities for plant conservation. However, information on neutral genetic diversity in a small number of marker loci can still be valuable because genomic data are not available to most rare plant species. In the hope of bridging the gap between conservation science and practice, we outline how conservation practitioners can more efficiently employ population genetic information in plant conservation. We first review the current knowledge about neutral genetic variation (NGV) and adaptive genetic variation (AGV) in seed plants, regarding both within‐population and among‐population components. We then introduce the estimates of among‐population genetic differentiation in quantitative traits (*Q*_ST_) and neutral markers (*F*_ST_) to plant biology and summarize conservation applications derived from *Q*_ST_–*F*_ST_ comparisons, particularly on how to capture most AGV and NGV on both in‐situ and ex‐situ programs. Based on a review of published studies, we found that, on average, two and four populations would be needed for woody perennials (*n* = 18) to capture 99% of NGV and AGV, respectively, whereas four populations would be needed in case of herbaceous perennials (*n* = 14). On average, *Q*_ST_ is about 3.6, 1.5, and 1.1 times greater than *F*_ST_ in woody plants, annuals, and herbaceous perennials, respectively. Hence, conservation and management policies or suggestions based solely on inference on *F*_ST_ could be misleading, particularly in woody species. To maximize the preservation of the maximum levels of both AGV and NGV, we suggest using maximum *Q*_ST_ rather than average *Q*_ST_. We recommend conservation managers and practitioners consider this when formulating further conservation and restoration plans for plant species, particularly woody species.

## Graphical abstract

One of the conservation applications derived from *Q*_ST_–*F*_ST_ comparisons is that they provide useful insights on how to capture most neutral (NGV) and adaptive (AGV) genetic variation on both in‐situ and ex‐situ programs. To capture 99% of NGV (based on *F*_ST_, a parameter of the degree of among‐population genetic differentiation) and AGV (based on *Q*_ST_, an *F*_ST_ analog for quantitative traits), two and four populations would be needed for perennial trees, respectively. For annuals, we would need four and six populations, respectively, whereas four populations would be needed for herbaceous perennials for both AGV and NGV. On average, *Q*_ST_ is approximately 3.6‐, 1.5‐, and 1.1‐fold greater than *F*_ST_ for woody plants, annuals, and herbaceous perennials, respectively. For both AGV and NGV, it is recommended to use the maximum *Q*_ST_ rather than the average *Q*_ST_ to best preserve the maximum level. For example, for the widespread tree *Populus balsamifera* in North America, two populations (*Ф*_ST_, an *F*_ST_ analog = 0.067) would be needed to capture 99% of NGV, whereas up to 25 populations would be targeted to maintain sufficient AGV (*Q*_ST_ = 0.832 for the bud set). Conservation managers and practitioners are encouraged to consider this when formulating further conservation and restoration plans for plant species, particularly woody species.

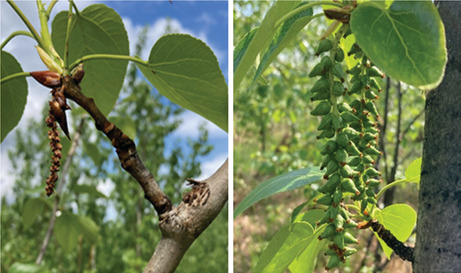

## 1 INTRODUCTION

Genetic diversity is a prerequisite for evolutionary change in all organisms; preservation of genetic diversity in a species likely increases its chances of surviving over evolutionary time when facing environmental changes. Plant evolutionary biologists, foresters, and conservation geneticists have long been interested in the genetic differences among populations and the degree to which these may reflect local adaptation (see Table 1 for the definition of population genetic terms cited in this mini review). This interest traces back to the common garden experiments of Turesson (1922) and the reciprocal transplants of Clausen et al. (1941). For decades, common garden and reciprocal transplant experiments have been instrumental in advancing our understanding of how natural selection shapes geographic phenotypic variation (reviewed in Flanagan et al., 2018; Sork, 2018). As putatively neutral molecular genetic markers (i.e., allozymes and DNA‐based dominant and codominant loci) became available, plant biologists were able to compare the levels of genetic diversity at single gene markers and the degree of divergence seen at phenotypic traits (De Kort et al., 2013; Leinonen et al., 2013; Marin et al., 2020; Reed & Frankham, 2001).

**TABLE 1:** Definitions of terms used in this mini review.

Term	Definition
Adaptation	A trait that increases the ability of a population or an organism to survive in its environment.
Allelic richness (*AR*)	A measurement of the number of alleles per locus with rarefaction adjusting for differences in sample sizes.
Balancing selection	A process in which more than one allele is maintained at a locus at a frequency higher than expected by chance. Balancing selection can come about due to overdominance (heterozygote advantage) or frequency‐dependent selection.
Broad‐sense heritability (*H* ^2^)	The ratio of total genetic variance to total phenotypic variance within a population.
Common garden experiment	A traditional experiment in which genotypes from different populations (provenances) are grown under a common environment to test the relative contribution of genetic and environmental variation on a given phenotypic trait.
Conservation genetics	A branch of (population) genetics aimed to reduce the risk of population and species extinctions and to design strategies for their preservation or restoration.
Conservation genomics	The use of genome‐scale data with the same aims of conservation genetics, i.e., ensuring the viability of populations and the biodiversity of living organisms.
*F*_ST_	The probability of identity by descent (*ibd*; describing the pair of homologous DNA sequences [for simplicity, *alleles*] carried by the gametes that produced it from a recent ancestor) resulting from population subdivision (independent of inbreeding within subdivisions); *F*_ST_ measures the probability of *ibd* of alleles within subpopulations relative to the total population.
*G*_ST_	The proportion of total genetic diversity found among populations averaged over all polymorphic loci; it is regarded as a multiallelic variant of Wright's *F*_ST_ (1951).
Gene diversity (*H*_e_)	Hardy–Weinberg expected heterozygosity both at monomorphic and polymorphic loci. The probability that an individual will be heterozygous at a given locus, based on allele frequencies at that locus.
Gene flow	The movement of alleles from one population to another population, which for plants is achieved by the transport of pollen and seeds by wind, water, or animals.
Genetic drift	A change in allele frequencies in a population over time resulting from a random sampling of gametes (i.e., error) to produce zygotes in the next generation and from chance variation in individuals' survival and/or reproductive success. Thus, it results in nonadaptive evolution.
Genetic markers	Any type of neutral (see below) genetic information (e.g., allozymes, amplified fragment length polymorphism, inter‐simple sequence repeats, microsatellites, DNA sequences [e.g., single nucleotide polymorphisms, SNPs]) that can be used to identify differences between individuals, populations, and/or species.
Isolation by distance	A process by which geographically restricted gene flow results in genetic differentiation being an increasing function of geographic distance.
Linkage disequilibrium	A state in which genes are combined in a dependent manner (i.e., linkage). It arises when genotypes at one locus within a population are non‐randomly distributed with respect to genotypes at another locus.
Local adaptation	A situation in which resident genotypes have a relatively higher fitness in their local environments than in other environments.
Narrow‐sense heritability (*h* ^2^)	The ratio of additive genetic variance to the phenotypic variance in a trait within a population.
Neutral	Molecular markers that do not affect fitness, i.e., individuals with different genotypes *A*_1_ *A*_1_ vs. *A*_1_ *A*_2_ have the same fitness.
Non‐additive genetic variation	Results from interactions between alleles at the same locus (dominance) or at different loci (epistasis).
Percentage of polymorphic loci (*%P*)	A measure used to quantify genetic diversity.
*Q*_ST_	The proportion of total additive genetic variance that is due to among‐population differences in a quantitative trait.
*Q*_ST_–*F*_ST_ comparison experiment	The comparison of the degree of genetic differentiation in quantitative traits (*Q*_ST_) with that in neutral molecular markers (*F*_ST_). This comparison allows the identification of a trait divergence caused by natural selection, as opposed to genetic drift.
Reciprocal transplant experiment	A traditional experimental approach in which living organisms from two different environments are reciprocally grown in their respective environments. If the phenotype of the transplanted individuals does not converge towards that of individuals in receiving population would be evidence for the strong genetic basis of the focal trait. The opposite outcome would be evidence for plasticity in determining the trait value.
Translocation	The deliberate (human‐mediated) transfer of plants (entire plants, seeds, or propagules) from an ex situ collection or a natural population to a new location, usually in the wild.

Applications of traditional marker‐based neutral genetic variation (NGV) to the conservation and restoration of plant species have been somewhat controversial due to the assumed evolutionary neutrality of used markers and their limitations to be informative about the adaptive potential (García‐Dorado & Caballero, 2021; Teixeira & Huber, 2021). Although levels of NGV might not be always predictive of adaptive genetic variation (AGV; Teixeira & Huber, 2021), it is possible that NGV under current conditions may become AGV under changed environmental conditions. However, NGV, largely corresponding to within‐population genetic variation from allozymes to nucleotide sequences—as reflected in the percentage of polymorphic loci (*%P*), allelic richness (*AR*), or gene diversity (Hardy–Weinberg expected heterozygosity, *H*_e_)—is considered a poor “proxy” of levels of AGV in quantitative traits (i.e., narrow‐ and broad‐sense heritabilities [*h* ^2^ and *H* ^2^]; Depardieu et al., 2020; Reed & Frankham, 2001).

The same applies to the relationship between measures of among‐population genetic differentiation (e.g., Merilä & Crnokrak, 2001). The comparison between *F*_ST_ ([Wright, 1951] or its analogs estimated from neutral genetic markers [Meirmans & Hedrick, 2010]; see Holsinger & Weir, 2009 for different definitions and interpretations of *F*_ST_) and *Q*_ST_ (*F*_ST_ analog for quantitative traits; Depardieu et al., 2020; Spitze, 1993), i.e., *Q*_ST_–*F*_ST_ comparisons or relationships, was formalized with the adoption of *Q*_ST_ in the 1990s. *Q*_ST_ creates an explicit prediction of the expectation for quantitative trait differentiation under neutrality (De Kort et al., 2013; Leinonen et al., 2013; Merilä & Crnokrak, 2001). Under the reasonable assumption that the genetic markers used commonly to estimate *F*_ST_ are neutral, the common finding that *Q*_ST_ > *F*_ST_ supports the view that the divergence of quantitative traits among populations exceeds neutral divergence and hence is predominantly driven by natural selection. Although *F*_ST_ is generally a poor predictor of *Q*_ST_, many researchers still assume that levels of NGV would be indicative of those of AGV (e.g., DeWoody et al., 2021; García‐Dorado & Caballero, 2021; Hamrick & Godt, 1996; Oostermeijer et al., 1994; Ottewell et al., 2016, but see Teixeira & Huber, 2021).

Although there is already an ongoing transition from conservation genetics to conservation genomics (Allendorf et al., 2010, 2022; Sork, 2018), conservation managers and practitioners need to continuously utilize information on NGV, if any, to support their decision making because genomic data are still scarce for many rare plant species. Comparative (i.e., *Q*_ST_–*F*_ST_ comparisons) and theoretical studies of NGV and AGV within and among populations in a variety of organisms are abundant in the literature (e.g., Hendry, 2002; Leinonen et al., 2013; Li et al., 2019; McKay & Latta, 2002; Reed & Frankham, 2001, 2003 and references therein). However, few studies have so far described or considered the application of *Q*_ST_–*F*_ST_ comparisons in the field of conservation biology (Reed & Frankham, 2003; but see Gravuer et al., 2005; McKay et al., 2001; Petit et al., 2001; Rodríguez‐Quilón et al., 2016).

On a different but related note, there have been increasing recommendations for lowering the gap between conservation science and practice (also coined as “the conservation genetics gap”, “the research‐implementation gap”, or “the science‐practice gap”; Britt et al., 2018; Dubois et al., 2019; Fabian et al., 2019; Holderegger et al., 2019; Taylor et al., 2017). It is generally agreed that conservation researchers should communicate with practitioners to integrate their genetic findings into conservation implementation (Chung et al., 2021; Ottewell et al., 2016). To achieve this, a generally and clearly written narrative covering *Q*_ST_–*F*_ST_ in seed plants might be needed to lower the threshold for plant conservation practitioners to employ population genetics information in conservation practice.

With this in mind, we first introduce the current knowledge about within‐population genetic variation and among‐population differentiation both in NGV and AGV in seed plants to highlight the distinction between the approaches used to identify the two types of genetic variation. Next, we introduce the known general application of *Q*_ST_–*F*_ST_ comparisons to plant biology. We also provide management suggestions as to how to capture germplasms (e.g., seeds) covering most AGV and NGV based on the analyses of molecular and quantitative trait data.

## 2 COMPARISON OF WITHIN‐POPULATION GENETIC VARIATION: NEUTRAL MARKERS VERSUS ADAPTIVE TRAITS

As neutral genetic markers reflect demographic processes (including past demographic histories) within local populations, they are informative for management and conservation purposes. Small populations are generally susceptible to the loss of NGV and less adaptive to novel environments due to the loss of AGV through genetic drift (Reed & Frankham, 2003). It is known that the degree of individuals' heterozygosity (estimated as the number of loci at which each individual is heterozygous) is often correlated with fitness (Oostermeijer et al., 1994; Reed & Frankham, 2003). Even when there is a real relationship between an individual's heterozygosity and fitness, this does not imply that there should be a relationship between *H*_e_ and *h* ^2^ at the population level. These two estimates are determined by somewhat different processes.

In a meta‐analysis of 71 (60 out of these with allozymes) published datasets, *H*_e_ was only weakly correlated with *h* ^2^ or *H* ^2^: *r* = 0.217 (−0.88 to 0.90, SD ± 0.433), indicating that neutral marker‐based measures only explain 4% of the variation in quantitative traits (Reed & Frankham, 2001). In addition, the correlation between allozyme‐based *H*_e_ and *h* ^2^ for 17 metric characters in seven populations of the annual *Phlox drummondii* was found to be highly variable, ranging from *r* = −0.714 to 0.355 (recalculated from Schwaegerle et al., 1986). Likewise, the correlation between microsatellite‐based *H*_e_ and *H* ^2^ estimated from five phenotypic traits in seven populations of the endangered herb *Psilopeganum sinense* ranged from *r* = −0.707 to 0.262 (Ye et al., 2014). Similar results revealing a weak correlation between NGV and AGV are available from other wild plant species as well. Examples include the rare perennial herb *Scabiosa canescens* and its common congener *S. columbaria* (allozyme‐based *H*_e_ vs. *H* ^2^; Waldmann & Andersson, 1998); the annual *Clarkia dudleyana* (allozyme‐based *H*_e_ vs. *CV*_G_ [coefficient of genetic variation of quantitative traits]; Podolsky, 2001), the annual *Hordeum spontaneum* (allozyme‐based *H*_e_ vs. *H* ^2^; Volis et al., 2005), and the selfing annual *Senecio vulgaris* (AFLP [amplified fragment length polymorphism]‐based *H*_e_ vs. *H* ^2^; Steinger et al., 2002).

The studies listed above suggest that NGV has a limited ability to predict AGV within populations. Reed and Frankham (2001) listed six factors that could be responsible for the low correlation between NGV and AGV, namely, differential selection, non‐additive genetic variation, different mutation rates (*μ*), low‐statistical power, environmental effects on quantitative characters, and impact of regulatory variation. In addition, various forms of natural selection affecting the level of neutral polymorphism at linked sites may also contribute to the lack of a relationship between NGV and AGV. The most dramatic effect on neutral variation occurs when beneficial alleles at loci contributing to AGV spread into a population, a process known as a “selective sweep” (Nielson, 2005; Stephan, 2019). Selective sweep can lead to a very large reduction of local *H*_e_ and *AR* along the chromosome segment (Kreitman, 2001). *H*_e_ and *AR* for non‐neighboring or unlinked neutral regions are likely not affected by such events (Nielson, 2005), because linkage disequilibrium between NGV and AGV decays gradually under the influence of recombination.

It should be noted that, however, invoking selective sweep as a factor that lowers the correlation between NGV and AGV could be problematic. The sweeping of one beneficial allele means that the AGV in that gene also disappears. Therefore, because AGV and NGV can be both high in the absence of a selective sweep, they can be both reduced after a sweep, and a positive correlation between AGV and NGV can be still maintained. Therefore, we need to ask whether there are other forms of natural selection in which NGV is lowered without reducing AGV. One such scenario, the hitchhiking effect of fluctuating selection, was provided by Barton (2000): fluctuating environment causing the adaptive alleles to oscillate between low and high frequencies, thus maintaining AGV without fixation or loss, is expected to reduce the levels of the surrounding NGV. The feasibility of such an evolutionary scenario is receiving growing attention, as fitness is indeed found to fluctuate rapidly and widely in natural populations (Bell, 2010; Messer et al., 2016) and population genomic studies have revealed seasonal oscillations of allele frequencies at a large number of sites (Bergland et al., 2014; Machado et al., 2021).

Under balancing selection, different alleles affecting fitness are maintained via heterozygote advantage, rare‐allele advantage, or temporally/spatially heterogeneous selection. By definition, such loci harbor high levels of AGV (Aguilar et al., 2004; Charlesworth, 2006). The level of NGV is also expected to be elevated at sites closely linked to the loci of stable balanced polymorphism (Charlesworth, 2006). However, only very closely neighboring neutral sites may experience such an increase in polymorphism because meiotic recombination quickly erodes linkage disequilibrium around the selected loci (Fijarczyk & Babik, 2015). This suggests that a high level of AGV can be maintained by balancing selection without a proportional increase in NGV on the genomic average. Considering this point, balancing selection could also contribute to the lack of a positive correlation between NGV and AGV. In sum, heterozygosity at adaptive and neutral loci is expected to be impacted by different evolutionary factors, which may explain why estimators of NGV are poor surrogates for AGV within plant populations.

## 3 COMPARISON OF AMONG‐POPULATION DIFFERENTIATION: NEUTRAL MARKERS VERSUS ADAPTIVE TRAITS

As sessile plants are subject to spatially divergent selection, elucidating the effects of local adaptation on population differentiation has become more important in light of adaptation to changing environments, including global climate change (Colautti et al., 2012; Ehrlich & Raven, 1969; Savolainen, 2011). A commonly used way to infer the impact of divergent selection on plant population differentiation is by comparing *Q*_ST_ (reflecting differentiation caused by both neutral and selective forces) versus *F*_ST_ estimates (reflecting differentiation due to neutral processes including genetic drift; Whitlock, 2008). The neutrality expectation depends on the assumption that mutation rates (*μ*) are substantially lower than migration rates (*m*; Hendry, 2002). Neutral markers having high *μ* (e.g., microsatellites) are not recommended to be used in *Q*_ST_–*F*_ST_ comparisons (Edelaar et al., 2011; Hendry, 2002), unless hypervariable loci are excluded (Li et al., 2019).

The *Q*_ST_–*F*_ST_ comparisons have already provided valuable insights into the evolutionary responses of plant traits to spatiotemporal environmental heterogeneity (Kremer et al., 1997; Leinonen et al., 2008, 2013; McKay & Latta, 2002; Merilä & Crnokrak, 2001; Savolainen et al., 2007; Volis et al., 2005). The *Q*_ST_–*F*_ST_ relationship can yield three different outcomes (Leinonen et al., 2008; Merilä & Crnokrak, 2001): *Q*_ST_ > *F*_ST_, *Q*_ST_ ≈ *F*_ST_, or *Q*_ST_ < *F*_ST_. First, if *Q*_ST_ > *F*_ST_, the observed trait differentiation exceeds neutral expectation and the observed differentiation is likely to have been caused by disruptive (divergent) selection. Second, if *Q*_ST_ ≈ *F*_ST_, trait differentiation is indistinguishable from the effects of drift, and thus, there is no evidence for selection (Lande, 1992). Finally, if *Q*_ST_ < *F*_ST_, trait divergence among populations is less than expected due to genetic drift alone probably under strong spatially uniform or stabilizing selection. The R package “driftsel” (Karhunen et al., 2013, 2014; Ovaskainen et al., 2011) can be used to differentiate between stabilizing selection, diversifying selection, and random genetic drift, allowing one to circumvent a lot of the problems with the traditional *Q*_ST_–*F*_ST_ comparisons.

Using several simple generalized linear models, Leinonen et al. (2008) carried out a meta‐analysis of 55 animal and plant studies that estimated *F*_ST_ and *Q*_ST_ from the same populations. They found a weak but significant positive correlation between *Q*_ST_ and *F*_ST_ (Spearman rank correlation, *r*_s_ = 0.39, *p* = .017) and that *Q*_ST_ > *F*_ST_ (*p* < .001), confirming the main conclusions of Merilä and Crnokrak (2001). Leinonen et al. (2008) suggested that genetic differentiation due to natural selection and local adaptation is the norm rather than the exception. The positive correlation between the degree of adaptive phenotypic divergence and differentiation at neutral loci is mainly caused by limited gene flow and enhanced local adaptation, a phenomenon known as “isolation by adaptation” (Nosil et al., 2007). Leinonen et al. (2008) further found that the study design (viz., wild, broad sense, and narrow sense), marker type (restriction fragment length polymorphisms, random amplified polymorphic DNAs, microsatellites, allozymes, and AFLPs), and trait type (morphological traits and life‐history traits) rarely explain any significant variance in the *Q*_ST_ data. They also pointed out two potential biases in finding that 70% of *Q*_ST_ values exceed the associated *F*_ST_ values: (i) a sampling bias due to the deliberate selection of populations from contrasting environments to be investigated, as well as focus on populations previously known to be phenotypically divergent; (ii) a publication bias favoring studies reporting *Q*_ST_ > *F*_ST_ outcomes, possibly because of difficulties interpreting *Q*_ST_ ≈ *F*_ST_ and *Q*_ST_ < *F*_ST_ patterns. *Q*_ST_ < *F*_ST_ could be due to canalization, which refers to a process or tendency in which “species genetic backgrounds share the same genetic constraints” (Lamy et al., 2012) representing “a fundamental feature of many developmental systems” (Hall et al., 2007). To partially distinguish canalization and uniform selection, Lamy et al. (2012) suggested “a bottom‐up approach” that combines information from *Q*_ST_–*F*_ST_ comparisons and phylogenetic reconstruction. For a given trait, if *Q*_ST_ < *F*_ST_ and phylogenetically closely related species occurring under different environmental conditions exhibit trait conservatism, then canalization could be inferred as an alternative to the classical uniform selection hypothesis (cf. figure 3 in Lamy et al., 2012). Well‐known examples of canalization in plants are leaf shape in *Arabidopsis thaliana* and cavitation resistance found in all *Pinus* species (Hall et al., 2007; Lamy et al., 2011).

The study by De Kort et al. (2013) was the first meta‐analysis of *Q*_ST_–*F*_ST_ comparisons exclusively focusing on plants. The authors compiled 51 entries representing 44 plant species from 18 families covering 17 entries for annuals, 19 for herbaceous perennials, and 15 for woody species. They found that average *Q*_ST_ values were significantly larger than the corresponding *F*_ST_ values (0.345 vs. 0.214, Wilcoxon signed‐rank test, *p* = .003; recalculated from original data from De Kort et al., 2013). The authors also found that the excess of *Q*_ST_ relative to *F*_ST_ was significantly negatively correlated with *F*_ST_ (*β* = −0.484, *p* < .01). A weak but positive overall relationship between pairwise *Q*_ST_ and *F*_ST_ values (*r*_s_ = 0.278, *p* = .048; *β* = 0.464, *p* = .003, recalculated from De Kort et al., 2013) suggests that *F*_ST_ in neutral markers could be to some degree predictive of *Q*_ST_ in quantitative traits. These correlations are what one would expect because (i) *Q*_ST_ reflects both neutral forces and natural selection caused by environmental differences and *F*_ST_ only measures neutral processes including genetic drift and gene flow, (ii) *Q*_ST_ and *F*_ST_ estimates are based on the same (among‐population) partition of total genetic variation, differing only in the data used in estimation—quantitative adaptive loci (the former) and neutral loci (the latter), and (iii) divergent selection that causes *Q*_ST_ could also lead to the increase of *F*_ST_ by restricting gene flow (“isolation by adaptation”; Nosil et al., 2007). In addition, De Kort et al. (2013) found a significant positive correlation between the average inter‐population distance and their *Q*_ST_–*F*_ST_ difference values (*p* < .05), suggesting that isolation by distance plays an important role in adaptive evolution. The authors' meta‐analysis suggests that plant species are generally differentiated by natural selection in various types of traits (viz., fitness [reproductive and physiological traits] and non‐fitness [biomass‐related and phenological traits] both in early life and in the adult stage). For example, the authors detected a larger *Q*_ST_–*F*_ST_ difference values for non‐fitness traits than for fitness traits, confirming the expectation that the former respond, in general, faster to directional selection than the latter (Leinonen et al., 2008; Merilä & Sheldon, 1999). Finally, De Kort et al. (2013) found slightly higher *Q*_ST_–*F*_ST_ difference values for annuals than perennials (0.143 vs. 0.123), but the difference was not significant. This can be viewed to be at odds with the prediction (De Kort et al., 2013) that perennials can respond to selection slower than annuals.

In closing, the differences in *F*_ST_ and *Q*_ST_ are products of the different evolutionary forces such as drift, gene flow, and selection (Slatkin, 1973), which can be further influenced by phenotypic plasticity, environmental maternal effects, non‐additive genetic interactions, pleiotropy, and possible differences in *μ* for *F*_ST_ and *Q*_ST_ (for more details see De Kort et al., 2013).

## 4 APPLICATION OF *Q*_ST_–*F*_ST_ COMPARISONS TO PLANT BIOLOGY

*Q*_ST_–*F*_ST_ comparisons have been used to estimate ecological and evolutionary processes in various plant species, including local adaptation, sexual selection, evolutionary stasis, human‐induced evolution, and artificial selection, among others. Perhaps, the most commonly studied issue has been to identify natural selection as a cause of broad‐scale clinal variation in morphological and life‐history traits (local adaptation; e.g., in *Campanulastrum americanum* [Prendeville et al., 2013], in *Helianthus maximiliani* [Kawakami et al., 2011], in two subspecies of *Antirrhinum majus* [Marin et al., 2020] or various tree species [Savolainen et al., 2007]). Regarding sexual selection, Yu et al. (2011) detected sex‐specific selection as the cause of the evolution of sexual dimorphism in *Silene latifolia*, while Lamy et al. (2011) identified selective constraints explaining phenotypic uniformity across species distributions in *Pinus pinaster*.

*Q*_ST_–*F*_ST_ comparisons have also been used to unravel human‐induced processes. Examples include the demonstration of how human‐induced habitat changes can either cause or impair adaptation (human‐induced evolution; e.g., *Thlaspi caerulescens* [Jiménez‐Ambriz et al., 2007] and *Arabidopsis halleri* [Meyer et al., 2010]) and of how selective breeding shapes diversification and population structuring of crop species (artificial selection; e.g., *Oryza sativa* [Sreejayan et al., 2011] and *Zea mays* [Pressoir & Berthaud, 2004]). By performing *Q*_ST_–*F*_ST_ comparisons between the invasive species' native and invasive ranges (biological invasions), several researchers have provided information on the evolution of invasiveness and the adaptive potential of invasive plant species (e.g., *Hypericum canariense* [Dlugosch & Parker, 2007], *Ambrosia artemisiifolia* [Chun et al., 2011], *Lythrum salicaria* [Chun et al., 2009], and *Geranium carolinianum* [Shirk & Hamrick, 2014]).

## 5 INSIGHTS INTO CONSERVATION AND RESTORATION DERIVED FROM *Q*_ST_–*F*_ST_ COMPARISONS

The *Q*_ST_–*F*_ST_ comparisons, along with geographic and environmental data, have been used to establish translocation schemes for population augmentation of rare plants (e.g., *Liatris scariosa* [Gravuer et al., 2005]). Furthermore, it has been suggested that setting conservation priorities should not be based only on neutral marker diversity and that *Q*_ST_–*F*_ST_ comparisons could be used to identify populations suitable for translocations (e.g., *Arabis fecunda* [McKay et al., 2001] and *Araucaria araucana* [Bekessy et al., 2003]). Conservation practitioners may also need information about how to capture most AGV and NGV based on known levels of NGV and AGV from population or conservation genetic studies. Because *F*_ST_ estimates are significantly lower in trees than in most herbaceous perennials and annuals, Chung et al. (2020) recommended that separate conservation genetic strategies should be designed for tree species and other plant species. Seeds of most tree species (which generally show low values of *F*_ST_) could be sourced from a few populations distributed across the species' range, whereas seeds of rare herbaceous species (often with high *F*_ST_ values) should be taken from many populations to capture the highly localized genetic diversity. Based on a small body of available data on seed plant species (De Kort et al., 2013; Lamy et al., 2012; Leinonen et al., 2013), *Q*_ST_ is on average higher than *F*_ST_ in common forest tree species, indicating that their quantitative traits have been subject to diversifying selection and local adaptation (Kremer et al., 1997; Savolainen et al., 2007). It has been suggested that more populations would be needed to preserve enough AGV for adaptively significant quantitative traits than for NGV, particularly in trees (Chung et al., 2020; Hamrick et al., 2006; McKay et al., 2001).

Population(s) to be protected in situ or to be sampled for seed banking purposes could be estimated using the following formulae: PNGV = 1 – *F*_ST_ (or *G*_ST_)^*N*^ for NGV, where PNGV = proportion of NGV captured by sampling, *N* = number of populations (Ceska et al., 1997; Hamrick et al., 2006) and PAGV = 1 – *Q*_ST_^*N*^ for AGV (J. D. Nason; P. Meirmans, pers. comms.), where PAGV = proportion of AGV captured by sampling. However, one should be aware that if there are more than two alleles per locus for the neutral markers, then *Q*_ST_ and *F*_ST_ are on different scales, and the formulae PAGV = 1 – *Q*_ST_^*N*^ and PNGV = 1 – *F*_ST_^*N*^ cannot be interpreted in the same way (J. D. Nason, pers. comm.). For multi‐allelic markers, it depends on *μ* whether this is problematic—for bi‐allelic single nucleotide polymorphisms this does not constitute a problem. Since *Ф*_ST_, the ratio of the among‐population variance component to total variance obtained based on genetic distances among alleles for the neutral markers, is conceptually similar to *Q*_ST_, it is advisable to use *Ф*_ST_ rather than *G*_ST_, *F*_ST_, or *θ* (Edelaar et al., 2011). The calculations for 99% capture of AGV and NGV can be the key to figuring out ideal sample sizes, especially when resources are limited. Based on the (recalculated) average values of De Kort et al. (2013) for *F*_ST_ and *Q*_ST_ (annuals, *n* = 19, 0.308 versus 0.451 [i.e., *Q*_ST_ is about 1.5 times greater than *F*_ST_]; herbaceous perennials, *n* = 14, 0.267 versus 0.299 [*Q*_ST_ is about 1.1 times greater]; woody perennials, *n* = 18, 0.074 versus 0.269 [*Q*_ST_ is about 3.6 times greater]), to capture 99% of NGV and AGV for woody perennials, only two and four populations would be needed using the abovementioned formulae, respectively. On the other hand, four populations of herbaceous perennials would be needed to secure 99% of NGV and AGV, respectively, because the average difference between *Q*_ST_ and *F*_ST_ is small (0.032). For annuals, four and six populations would be needed to secure 99% of NGV and AGV, respectively.

**FIGURE 1:**
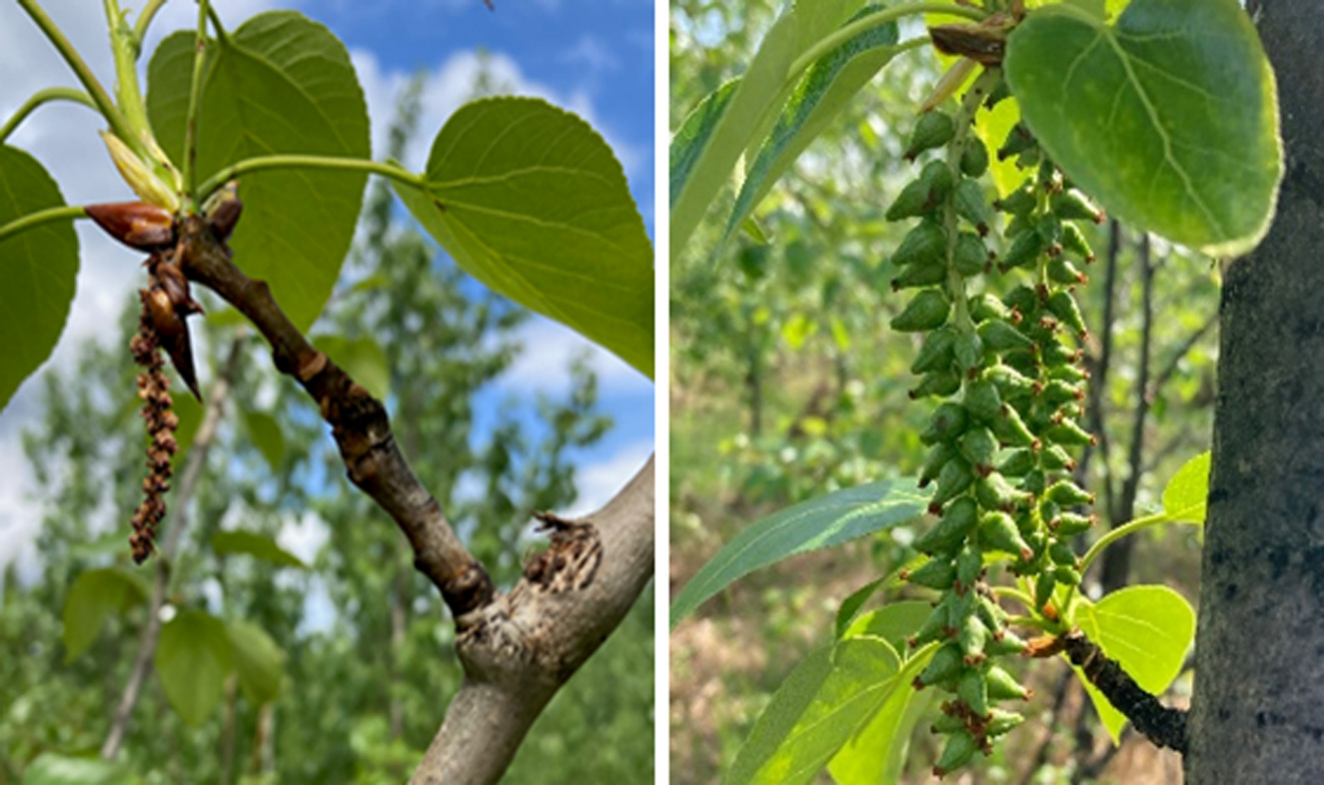
Photographic images of the male (left) and female (right) catkins of balsam poplar (*Populus balsamifera*), a fast‐growing and widespread hardwood in northernmost North America. Photos were taken by Matthew Olson at Texas Tech University.

We applied the above‐mentioned approach to the widespread tree *Populus balsamifera* (Figure 1) for which adequate genetic data have been obtained; Keller et al. (2011) reported a mean *Ф*_ST_ value of 0.067 estimated from 310 nuclear SNP loci and a mean *Q*_ST_ value of 0.421 (range = 0.127–0.832) obtained from 13 ecophysiological and phenological traits originating from 20 populations across North America. Two populations from this tree species would be needed to capture 99% of NGV using the above formula. When we apply the mean *Q*_ST_ value to the formula, at least six populations would be necessary to capture the same level of AGV. However, the value of *Q*_ST_ depends on the trait under consideration: for traits with a high *Q*_ST_, more populations should be sampled than for traits with a low *Q*_ST_. Application of too low values of *Q*_ST_ for this equation would lead to an underestimation of the number of populations needed to preserve the desired level of genetic variation. Given this, it would be wiser not to use the average *Q*_ST_ but the maximum *Q*_ST_. Thus, as in the case of the *P. balsamifera Q*_ST_ = 0.832 for the bud set, then up to 25 populations would be needed to be targeted to maintain enough AGV. Of course, this does not mean that NGV is not important; there is probably a reservoir of genetic variation in every population that is neutral now but that may become selectively important if environmental conditions change. Furthermore, NGV can be very informative about the populations' past demography which is often of interest in conservation biology (Allendorf, 2017; DeWoody et al., 2021; Frankham, 2015; García‐Dorado & Caballero, 2021).

The application of the above formulae to plants with different life forms, as well as the example of *Populus balsamifera*, suggests that conservation and management policies or actions based solely on *F*_ST_ could potentially be misleading. Again, these findings stress that guidelines and conservation genetic strategies should be designed based on genetic information on both NGV and AGV for tree and herbaceous (whether perennial or annual) species. Following the reasoning laid out above, managers, or practitioners should design restoration and conservation strategies by knowing that, on average, *Q*_ST_ is about 3.6, 1.5, and 1.1 times greater than *F*_ST_ in woody plants, annuals, and herbaceous perennials, respectively.

As *F*_ST_ appears to be more closely related to AGV than within‐population genetic diversity metrics (e.g., *H*_e_, *%P*, or *AR*), the former should be considered as a more predictable parameter for plant conservation and restoration purposes; estimating the value of *F*_ST_ (i.e., low, moderate, or high) is important for prioritizing populations for both in situ and ex situ collection and for identifying appropriate sources for reintroductions (Chung et al., 2021; Hamrick & Godt, 1996; Ottewell et al., 2016). Thus, the importance of the proper consideration of *F*_ST_ information (and *Q*_ST_, if available) in conservation management cannot be overstated, particularly when it comes to annuals and herbaceous perennials.

## 6 CONCLUSIONS AND PERSPECTIVE

Within‐population genetic variation, both natural and restored, is crucial for the response to short‐term environmental stresses and long‐term evolutionary change. Although the levels of *H*_e_ are often correlated with fitness (Oostermeijer et al., 1994; Reed & Frankham, 2003; Szulkin et al., 2010), *H*_e_ of NGV is poorly correlated with heritability (*h* ^2^ or *H* ^2^) of quantitative traits (AGV). As discussed above, the relationship of *H*_e_ to *h* ^2^ or *H* ^2^ is often very weak, while the relationship between *F*_ST_ and *Q*_ST_ is comparatively stronger; thus, *F*_ST_ could be considered a weak proxy of *Q*_ST_. However, whenever logistically possible, common garden and/or transplant studies are strongly recommended to quantify patterns of adaptive genetic variation and differentiation (Capblancq et al., 2020; de Villemereuil et al., 2016; Sork, 2018). The most comprehensive studies conducted so far are generally those carried out with many commercially important tree species (e.g., eucalypts, oaks, poplars, pines, and spruces), and plants with well‐adapted genotypes are already used to replant clear‐cut areas (Depardieu et al., 2020). Nevertheless, more studies on *Q*_ST_–*F*_ST_ comparisons are needed, particularly on rare woody species and common herbaceous species, to avoid biased inference, as well as to balance entries among the different life forms. With a larger dataset, one could also expect some generalizations to emerge concerning the *Q*_ST_–*F*_ST_ relationships regarding life history characteristics and morphological/anatomical traits. Such generalizations could aid conservation managers and practitioners in using neutral *F*_ST_ estimates to predict approximate *Q*_ST_ values and aid the conservation and restoration of plant species. Multiple approaches, including molecular markers (NGV), quantitative traits, and/or quantitative trait loci coding for traits and contemporary genome‐wide association approaches in the context of a common garden experiment, and environmental variation (e.g., designation of climatic zonation) are needed to gain comprehensive insights into conservation of herbs and trees (de Villemereuil et al., 2016; Rodríguez‐Quilón et al., 2016; Sork, 2018).

## AUTHOR CONTRIBUTIONS

**Mi Yoon Chung:** Conceptualization (lead); funding acquisition (lead); project administration (lead); writing – original draft (supporting); writing – review and editing (supporting). **Juha Merilä:** Conceptualization (supporting); writing – review and editing (equal). **Yuseob Kim:** Writing – review and editing (equal). **Kangshan Mao:** Writing – review and editing (equal). **Jordi López‐Pujol:** Writing – review and editing (equal). **Myong Gi Chung:** Conceptualization (lead); project administration (lead); writing – original draft (lead); writing – review and editing (lead).

## FUNDING INFORMATION

This work was supported by the research fund of Chungnam National University, the Republic of Korea to MYC.

## CONFLICT OF INTEREST STATEMENT

All the authors state that there is no conflict of interest.

## Data Availability

There was no new data created or analyzed for this manuscript.

## References

[ece39926-bib-0001] Aguilar, A. , Roemer, G. , Debenham, S. , Binns, M. , Garcelon, D. , & Wayne, R. K. (2004). High MHC diversity maintained by balancing selection in an otherwise genetically monomorphic mammal. Proceedings of the National Academy of Sciences of the United States of America, 101, 3490–3494. 10.1073/pnas.0306582101 14990802 PMC373489

[ece39926-bib-0002] Allendorf, F. W. (2017). Genetics and the conservation of natural populations: Allozymes to genomes. Molecular Ecology, 26, 420–430. 10.1111/mec.13948 27933683

[ece39926-bib-0003] Allendorf, F. W. , Funk, W. C. , Aitken, S. N. , Byrne, M. , & Luikart, G. (2022). Conservation and the genomics of populations (3rd ed., p. 746). Oxford University Press. 10.1111/eva.13499 PMC9753819 36540641

[ece39926-bib-0004] Allendorf, F. W. , Hohenlohe, P. A. , & Luikart, G. (2010). Genomics and the future of conservation genetics. Nature Reviews Genetics, 11, 697–709. 10.1038/nrg2844 20847747

[ece39926-bib-0092] Barton, N. H. (2000). Genetic hitchhiking. Philosophical Transactions of the Royal Society of London Series B: Biological Sciences, 355, 1553–1562. 10.1098/rstb.2000.0716 11127900 PMC1692896

[ece39926-bib-0005] Bekessy, S. A. , Ennos, R. A. , Burgman, M. A. , Newton, A. C. , & Ades, P. K. (2003). Neutral DNA markers fail to detect genetic divergence in an ecologically important trait. Biological Conservation, 110, 267–275.

[ece39926-bib-0006] Bell, G. (2010). Fluctuating selection: The perpetual renewal of adaptation in variable environments. Philosophical Transactions of the Royal Society B: Biological Sciences, 365, 87–97. 10.1098/rstb.2009.0150 PMC2842698 20008388

[ece39926-bib-0007] Bergland, A. O. , Behrman, E. L. , O'Brien, K. R. , Schmidt, P. S. , & Petrov, D. A. (2014). Genomic evidence of rapid and stable adaptive oscillations over seasonal time scales in *Drosophila* . PLoS Genetics, 10, e1004775. 10.1371/journal.pgen.1004775 25375361 PMC4222749

[ece39926-bib-0008] Britt, M. , Haworth, S. E. , Johnson, J. B. , Martchenko, D. , & Shafer, A. B. (2018). The importance of non‐academic coauthors in bridging the conservation genetics gap. Biological Conservation, 218, 118–123. 10.1016/j.biocon.2017.12.019

[ece39926-bib-0009] Capblancq, T. , Fitzpatrick, M. C. , Bay, R. A. , Exposito‐Alonso, M. , & Keller, S. R. (2020). Genomic prediction of (mal)adaptation across current and future climatic landscapes. Annual Review of Ecology, Evolution, and Systematics, 51, 245–269. 10.1146/annurev-ecolsys-020720-042553

[ece39926-bib-0010] Ceska, J. F. , Affolter, J. M. , & Hamrick, J. L. (1997). Developing a sampling strategy for *Baptisia arachnifera* based on allozyme diversity. Conservation Biology, 11, 1133–1139. 10.1046/j.1523-1739.1997.95527.x

[ece39926-bib-0011] Charlesworth, D. (2006). Balancing selection and its effects on sequences in nearby genome regions. PLoS Genetics, 2(4), e64. 10.1371/journal.pgen.0020064 16683038 PMC1449905

[ece39926-bib-0012] Chun, Y. J. , Le Corre, V. , & Bretagnolle, F. (2011). Adaptive divergence for a fitness‐related trait among invasive *Ambrosia artemisiifolia* populations in France. Molecular Ecology, 20, 1378–1388. 10.1111/j.1365-294X.2011.05013.x 21306459

[ece39926-bib-0013] Chun, Y. J. , Nason, J. D. , & Moloney, K. A. (2009). Comparison of quantitative and molecular genetic variation of native vs. invasive populations of purple loosestrife (*Lythrum salicaria* L., Lythraceae). Molecular Ecology, 18, 3020–3035. 10.1111/j.1365-294X.2009.04254.x 19548895

[ece39926-bib-0014] Chung, M. Y. , Son, S. , Herrando‐Moraira, S. , Tang, C. Q. , Maki, M. , Kim, Y.‐D. , López‐Pujol, J. , Hamrick, J. L. , & Chung, M. G. (2020). Incorporating differences between genetic diversity of trees and herbaceous plants in conservation strategies. Conservation Biology, 34, 1142–1151. 10.1111/cobi.13467 31994789

[ece39926-bib-0015] Chung, M. Y. , Son, S. , López‐Pujol, J. , Mao, K. , & Chung, M. G. (2021). Plant conservation practitioners can benefit from neutral genetic diversity. Diversity, 13, 552. 10.3390/d13110552

[ece39926-bib-0016] Clausen, J. , Keck, D. D. , & Hiesey, W. M. (1941). Experimental studies on the nature of species I. Effect of varied environments on western North American plants (Carnegie Institution of Washington Publication, 520). Carnegie Institution of Washington.

[ece39926-bib-0017] Colautti, R. I. , Lee, C. R. , & Mitchell‐Olds, T. (2012). Origin, fate, and architecture of ecologically relevant genetic variation. Current Opinion in Plant Biology, 15, 199–204. 10.1016/j.pbi.2012.01.016 22341792 PMC3413448

[ece39926-bib-0018] De Kort, H. , Vandepitte, K. , & Honnay, O. (2013). A meta‐analysis of the effects of plant traits and geographical scale on the magnitude of adaptive differentiation as measured by the difference between *Q* _ST_ and *F* _ST_ . Evolutionary Ecology, 27, 1081–1097. 10.1007/s10682-012-9624-9

[ece39926-bib-0019] de Villemereuil, P. , Gaggiotti, O. E. , Mouterde, M. , & Till‐Bottraud, I. (2016). Common garden experiments in the genomic era: New perspectives and opportunities. Heredity, 116, 249–254. 10.1038/hdy.2015.93 26486610 PMC4806574

[ece39926-bib-0020] Depardieu, C. , Girardin, M. P. , Nadeau, S. , Lenz, P. , Bousquet, J. , & Isabel, P. (2020). Adaptive genetic variation to drought in a widely distributed conifer suggests a potential for increasing forest resilience in a drying climate. New Phytologist, 227, 427–439. 10.1111/nph.16551 32173867 PMC7317761

[ece39926-bib-0021] DeWoody, J. A. , Harder, A. M. , Mathur, S. , & Willoughby, J. R. (2021). The long‐standing significance of genetic diversity in conservation. Molecular Ecology, 30, 4147–4154. 10.1111/mec.16051 34191374

[ece39926-bib-0022] Dlugosch, K. M. , & Parker, I. M. (2007). Molecular and quantitative trait variation across the native range of the invasive species *Hypericum canariense*: Evidence for ancient patterns of colonization via pre‐adaptation? Molecular Ecology, 16, 4269–4283. 10.1111/j.1365-294X.2007.03508.x 17850270

[ece39926-bib-0023] Dubois, N. S. , Gomez, A. , Carlson, S. , & Russell, D. (2019). Bridging the research‐implementation gap requires engagement from practitioners. Conservation Science and Practice, 2, e134. 10.1111/csp2.134

[ece39926-bib-0024] Edelaar, P. , Burraco, P. , & Gomez‐Mestre, I. (2011). Comparisons between *Q* _ST_ and *F* _ST_—How wrong have we been? Molecular Ecology, 20, 4830–4839. 10.1111/j.1365-294X.2011.05333.x 22060729

[ece39926-bib-0025] Ehrlich, P. R. , & Raven, P. H. (1969). Differentiation of populations. Science, 165, 1228–1232. 10.1126/science.165.3899.1228 5803535

[ece39926-bib-0026] Fabian, Y. , Bollmann, K. , Brang, P. , Hein, C. , Olschewski, R. , Rigling, A. , Stofer, S. , & Holderegger, R. (2019). How to close the science‐practice gap in nature conservation? Information sources used by practitioners. Biological Conservation, 235, 93–101. 10.1016/j.biocon.2019.04.011

[ece39926-bib-0027] Fijarczyk, A. , & Babik, W. (2015). Detecting balancing selection in genomes: Limits and prospects. Molecular Ecology, 24, 3529–3545. 10.1111/mec.13226 25943689

[ece39926-bib-0028] Flanagan, S. P. , Forester, B. R. , Latch, E. K. , Aitken, S. N. , & Hoban, S. (2018). Guidelines for planning genomic assessment and monitoring of locally adaptive variation to inform species conservation. Evolutionary Applications, 11, 1035–1052. 10.1111/eva.12569 30026796 PMC6050180

[ece39926-bib-0029] Frankham, R. (2015). Genetic rescue of small inbred populations: Meta‐analysis reveals large and consistent benefits of gene flow. Molecular Ecology, 24, 2610–2618. 10.1111/mec.13139 25740414

[ece39926-bib-0030] García‐Dorado, A. , & Caballero, A. (2021). Neutral genetic diversity as a useful tool for conservation biology. Conservation Genetics, 22, 541–545. 10.1007/s10592-021-01384-9-9

[ece39926-bib-0031] Gravuer, K. , von Wettberg, E. , & Schmitt, J. (2005). Population differentiation and genetic variation inform translocation decisions for *Liatris scariosa* var. *novae‐angliae*, a rare New England grassland perennial. Biological Conservation, 124, 155–167. 10.1016/j.biocon.2005.01.021

[ece39926-bib-0032] Hall, M. C. , Dworkin, I. , Ungerer, M. C. , & Purugganan, M. P. (2007). Genetics of microenvironmental canalization in *Arabidopsis thaliana* . Proceedings of the National Academy of Sciences of the United States of America, 104, 13717–13722. 10.1073/pnas.0701936104 17698961 PMC1959448

[ece39926-bib-0033] Hamrick, J. L. , & Godt, M. J. W. (1996). Conservation genetics of endemic plant species. In J. C. Avise & J. L. Hamrick (Eds.), Conservation genetics: Case histories from nature (pp. 281–304). Chapman & Hall.

[ece39926-bib-0034] Hamrick, J. L. , Godt, M. J. W. , & Gonzales, E. (2006). Conservation of genetic diversity in old‐growth forest communities of the southeastern United States. Applied Vegetation Science, 9, 51–57. 10.1111/j.1654-109X.2006.tb00655.x

[ece39926-bib-0035] Hendry, A. P. (2002). *Q*st > = ≠< *F*st? Trends in Ecology & Evolution, 17, 502. 10.1016/S0169-5347(02)02603-4

[ece39926-bib-0036] Holderegger, R. , Balkenhol, N. , Bolliger, J. , Engler, J. O. , Gugerli, F. , Hochkirch, A. , Nowak, C. , Segelbacher, G. , Wider, A. , & Zachos, F. E. (2019). Conservation genetics: Linking science with practice. Molecular Ecology, 28, 3848–3856. 10.1111/mec.15202 31392753

[ece39926-bib-0037] Holsinger, K. E. , & Weir, B. S. (2009). Genetics in geographically structured populations: Defining, estimating and interpreting *F* _ST_ . Nature Reviews Genetics, 10, 639–650. 10.1038/nrg2611 PMC4687486 19687804

[ece39926-bib-0038] Jiménez‐Ambriz, G. , Petit, C. , Bourrié, I. , Dubois, S. , Olivieri, I. , & Ronce, O. (2007). Life history variation in the heavy metal tolerant plant *Thlaspi caerulescens* growing in a network of contaminated and noncontaminated sites in southern France: Role of gene flow, selection and phenotypic plasticity. New Phytologist, 173, 199–215. 10.1111/j.1469-8137.2006.01923.x 17176406

[ece39926-bib-0039] Karhunen, M. , Merilä, J. , Leinonen, T. , Cano, J. M. , & Ovaskainen, O. (2013). DRIFTSEL: An R package for detecting signals of natural selection in quantitative traits. Molecular Ecology Resources, 13, 746–754. 10.1111/1755-0998.12111 23656704

[ece39926-bib-0040] Karhunen, M. , Ovaskainen, O. , Herczeg, G. , & Merilä, J. (2014). Bringing habitat information into statistical tests of local adaptation in quantitative traits: A case study of nine‐spined sticklebacks. Evolution, 68, 559–568. 10.1111/evo.12268 24117061

[ece39926-bib-0041] Kawakami, T. , Morgan, T. J. , Nippert, J. B. , Ocheltree, T. W. , Keith, R. , Dhakal, P. , & Ungerer, M. C. (2011). Natural selection drives clinal life history patterns in the perennial sunflower species, *Helianthus maximiliani* . Molecular Ecology, 20, 2318–2328. 10.1111/j.1365-294X.2011.05105.x 21521394

[ece39926-bib-0042] Keller, S. R. , Soolanayakanahally, R. Y. , Guy, R. D. , Silim, S. N. , Olson, M. S. , & Tiffin, P. (2011). Climate‐driven local adaptation of ecophysiology and phenology in balsam poplar, *Populus balsamifera* L. (Salicaceae). American Journal of Botany, 98, 99–108. 10.3732/ajb.1000317 21613088

[ece39926-bib-0043] Kreitman, M. (2001). Selective sweep. In S. Brenner & J. H. Miller (Eds.), Encyclopedia of genetics (pp. 1803–1804). Academic Press.

[ece39926-bib-0044] Kremer, A. , Zanetto, A. , & Ducousso, A. (1997). Multilocus and multitrait measures of differentiation for gene markers and phenotypic traits. Genetics, 14, 1229–1241. 10.1093/genetics/145.4.1229 PMC1207889 9093871

[ece39926-bib-0045] Lamy, J.‐B. , Bouffier, L. , Burlett, R. , Plomion, C. , Cochard, H. , & Delzon, S. (2011). Uniform selection as a primary force reducing population genetic differentiation of cavitation resistance across a species range. PLoS One, 6(8), e23476. 10.1371/journal.pone.0023476 21858137 PMC3155568

[ece39926-bib-0046] Lamy, J.‐B. , Plomion, C. , Kremer, A. , & Delzon, S. (2012). *Q* _ST_ < *F* _ST_ as a signature of canalization. Molecular Ecology, 21, 5646–5655. 10.1111/mec.12017 23110372

[ece39926-bib-0047] Lande, R. (1992). Neutral theory of quantitative genetic variance in an island model with local extinction and recolonization. Evolution, 46, 381–389. 10.1111/j.1558-5646.1992.tb02046.x 28564025

[ece39926-bib-0048] Leinonen, T. , McCairns, R. J. S. , O'Hara, R. B. , & Merilä, J. (2013). *Q* _ST_–*F* _ST_ comparisons: Evolutionary and ecological insights from genomic heterogeneity. Nature Reviews Genetics, 14, 179–190. 10.1038/nrg3395 23381120

[ece39926-bib-0049] Leinonen, T. , O'Hara, R. B. , Cano, J. M. , & Merilä, J. (2008). Comparative studies of quantitative trait and neutral marker divergence: A meta‐analysis. Journal of Evolutionary Biology, 21, 1–17. 10.1111/j.1420-9101.2007.01445.x 18028355

[ece39926-bib-0050] Li, Z. , Löytynoja, A. , Fraimout, A. , & Merilä, J. (2019). Effects of marker type and filtering criteria on *Q* _ST_‐*F* _ST_ comparisons. Royal Society Open Science, 6(11), 190666. 10.1098/rsos.190666 31827824 PMC6894560

[ece39926-bib-0051] Machado, H. E. , Bergland, A. O. , Taylor, R. , Tilk, S. , Behrman, E. , Dyer, K. , Fabian, D. K. , Flatt, T. , González, J. , Karasov, T. , Kim, B. , Kozeretska, I. , Lazzaro, B. P. , Merritt, T. J. S. , Pool, J. E. , O'Brien, K. , Rajpurohit, S. , Roy, P. R. , Schaeffer, S. W. , … Petrov, D. A. (2021). Broad geographic sampling reveals the shared basis and environmental correlates of seasonal adaptation in *Drosophila* . eLife, 10, e67577. 10.7554/eLife.67577 34155971 PMC8248982

[ece39926-bib-0052] Marin, S. , Gibert, A. , Archambeau, J. , Bonhomme, V. , Lascoste, M. , & Pujol, B. (2020). Potential adaptive divergence between subspecies and populations of snapdragon plants inferred from *Q* _ST_–*F* _ST_ comparisons. Molecular Ecology, 29, 3010–3021. 10.1111/mec.15546 32652730 PMC7540467

[ece39926-bib-0053] McKay, J. K. , Bishop, J. G. , Lin, J.‐Z. , Richards, J. H. , Sala, A. , & Mitchell‐Olds, T. (2001). Local adaptation across a climatic gradient despite small effective population size in the rare sapphire rockcress. Proceedings of the Royal Society B: Biological Sciences, 268, 1715–1721. 10.1098/rspb.2001.1715 PMC1088799 11506685

[ece39926-bib-0054] McKay, J. K. , & Latta, R. G. (2002). Adaptive population divergence: Markers, QTL and traits. Trends in Ecology & Evolution, 17, 285–291. 10.1016/S0169-5347(02)02478-3

[ece39926-bib-0055] Meirmans, P. , & Hedrick, P. W. (2010). Assessing population structure: *F* _ST_ and related measures. Molecular Ecology, 11, 5–18. 10.1111/j.1755-0998.2010.02927.x 21429096

[ece39926-bib-0056] Merilä, J. , & Crnokrak, P. (2001). Comparison of genetic differentiation at marker loci and quantitative traits. Journal of Evolutionary Biology, 14, 892–903.

[ece39926-bib-0057] Merilä, J. , & Sheldon, B. C. (1999). Genetic architecture of fitness and nonfitness traits: Empirical patterns and development of ideas. Heredity, 83, 103–109. 10.1046/j.1365-2540.1999.00585.x 10469197

[ece39926-bib-0058] Messer, P. W. , Ellner, S. P. , & Hairston, N. G., Jr. (2016). Can population genetics adapt to rapid evolution? Trends in Genetics, 32, 408–418. 10.1016/j.tig.2016.04.005 27185237

[ece39926-bib-0059] Meyer, C. L. , Kostecka, A. A. , Saumitou‐Laprade, P. , Créach, A. , Castric, V. , Pauwels, M. , & Frérot, H. (2010). Variability of zinc tolerance among and within populations of the pseudometallophyte species *Arabidopsis halleri* and possible role of directional selection. New Phytologist, 185, 130–142. 10.1111/j.1469-8137.2009.03062.x 19863732

[ece39926-bib-0060] Nielson, R. (2005). Molecular signatures of natural selection. Annual Review of Genetics, 39, 197–218. 10.1146/annurev.genet.39.073003.112420 16285858

[ece39926-bib-0061] Nosil, P. , Egan, S. P. , & Daniel, J. (2007). Heterogeneous genomic differentiation between walking‐stick ecotypes: “Isolation by adaptation” and multiple roles for divergent selection. Evolution, 62, 316–336. 10.1111/j.1558-5646.2007.00299.x 17999721

[ece39926-bib-0062] Oostermeijer, J. G. B. , van Fijek, M. W. , & den Nijs, J. C. M. (1994). Offspring fitness in relation to population size and genetic variation in the rare perennial plant species *Gentiana pneumonanthe* . Oecologia, 97, 289–296. 10.1007/BF00317317 28313622

[ece39926-bib-0063] Ottewell, K. M. , Bickerton, D. C. , Byrne, M. , & Lowe, A. J. (2016). Bridging the gap: A genetic assessment framework for population‐level threatened plant conservation prioritization and decision‐making. Diversity and Distributions, 22, 174–188. 10.1111/ddi.12387

[ece39926-bib-0064] Ovaskainen, O. , Karhunen, M. , Zheng, C. Z. , Arias, J. M. C. , & Merilä, J. (2011). A new method to uncover signatures of divergent and stabilizing selection in quantitative traits. Genetics, 189, 621–632. 10.1534/genetics.111.129387 21840853 PMC3189809

[ece39926-bib-0065] Petit, C. , Fréville, H. , Mignot, A. , Colas, B. , Riba, M. , Imbert, E. , Hurtrez‐Boussés, I. , Virevaire, M. , & Olivieri, I. (2001). Gene flow and local adaptation in two endemic plant species. Biological Conservation, 100, 21–34. 10.1016/S0006-3207(00)00204-4

[ece39926-bib-0066] Podolsky, R. H. (2001). Genetic variation for morphological and allozyme variation in relation to population size in *Clarkia dudleyana*, an endemic annual. Conservation Biology, 15, 412–423. 10.1046/j.1523-1739.2001.015002412.x

[ece39926-bib-0067] Prendeville, H. R. , Barnard‐Kubow, K. , Dai, C. , Barringer, B. C. , & Galloway, L. F. (2013). Clinal variation for only some phenological traits across a species range. Oecologia, 173, 421–430. 10.1007/s00442-013-2630-y 23474838

[ece39926-bib-0068] Pressoir, G. , & Berthaud, J. (2004). Population structure and strong divergent selection shape phenotypic diversification in maize landraces. Heredity, 92, 95–101. 10.1038/sj.hdy.6800388 14666128

[ece39926-bib-0069] Reed, D. H. , & Frankham, R. (2001). How closely correlated are molecular and quantitative measures of genetic variation? A meta‐analysis. Evolution, 55, 1095–1103. 10.1111/j.0014-3820.2001.tb00629.x 11475045

[ece39926-bib-0070] Reed, D. H. , & Frankham, R. (2003). Correlation between fitness and genetic diversity. Conservation Biology, 17, 230–237.

[ece39926-bib-0071] Rodríguez‐Quilón, I. , Santos‐del‐Blanco, L. , Serra‐Varela, M. J. , Koskela, J. , González‐Martínez, S. C. , & Alía, R. (2016). Capturing neutral and adaptive genetic diversity for conservation in a highly structured tree species. Ecological Applications, 26, 2254–2266. 10.1002/eap.1361 27755736

[ece39926-bib-0072] Savolainen, O. (2011). The genomic basis of local climatic adaptation. Science, 334, 49–50. 10.1126/science.1213788 21980101

[ece39926-bib-0073] Savolainen, O. , Pyhäjärvi, T. , & Knürr, T. (2007). Gene flow and local adaptation in trees. Annual Review of Ecology, Evolution, and Systematics, 38, 595–619. 10.1146/annurev.ecolsys.38.091206.095646

[ece39926-bib-0074] Schwaegerle, K. E. K. , Garbutt, K. , & Bazzaz, F. A. (1986). Differentiation among nine populations of *Phlox*. I. Electrophoretic and quantitative variation. Evolution, 40, 506–517. 10.1111/j.1558-5646.1986.tb00503.x 28556316

[ece39926-bib-0075] Shirk, R. Y. , & Hamrick, J. L. (2014). Multivariate adaptation but no increase in competitive ability in invasive *Geranium carolinianum* L. (Geraniaceae). Evolution, 68, 2945–2959. 10.1111/evo.12474 24931621

[ece39926-bib-0076] Slatkin, M. (1973). Gene flow and selection in a cline. Genetics, 75, 733–756. 10.1093/genetics/75.4.733 4778791 PMC1213045

[ece39926-bib-0077] Sork, V. L. (2018). Genomic studies of local adaptation in natural plant populations. Journal of Heredity, 109, 3–15. 10.1093/jhered/esx091 29045754

[ece39926-bib-0078] Spitze, K. (1993). Population structure in *Daphnia obtusa*: Quantitative genetic and allozyme variation. Genetics, 135, 67–374. 10.1093/genetics/135.2.367 PMC1205642 8244001

[ece39926-bib-0079] Sreejayan, N. , Kumar, U. S. , Varghese, G. , Jacob, T. M. P. , & Thomas, G. (2011). Stratification and population structure of the genetic resources of ancient medicinal rice (*Oryza sativa* L.) landrace Njavara. Genetic Resources and Crop Evolution, 58, 697–711. 10.1007/s10722-010-9613-1

[ece39926-bib-0080] Steinger, T. , Haldimann, P. , Leiss, K. , & Müller‐Schärer, H. (2002). Does natural selection promote population divergence? A comparative analysis of population structure using amplified fragment length polymorphism markers and quantitative traits. Molecular Ecology, 11, 2583–2590. 10.1046/j.1365-294x.2002.01653.x 12453241

[ece39926-bib-0081] Stephan, W. (2019). Selective sweeps. Genetics, 211, 5–13. 10.1534/genetics.118.301319 30626638 PMC6325696

[ece39926-bib-0082] Szulkin, M. , Bierne, N. , & David, P. (2010). Heterozygosity‐fitness correlations: A time for reappraisal. Evolution, 64, 1202–1217. 10.1111/j.1558-5646.2010.00966.x 20148954

[ece39926-bib-0083] Taylor, H. R. , Dussex, N. , & van Heezik, Y. (2017). Bridging the conservation genetics gap by identifying barriers to implementation for conservation practitioners. Global Ecology and Conservation, 10, 231–242. 10.1016/j.gecco.2017.04.001

[ece39926-bib-0084] Teixeira, J. C. , & Huber, C. D. (2021). The inflated significance of neutral genetic diversity in conservation genetics. Proceedings of the National Academy of Sciences of the United States of America, 118(10), e2015096118. 10.1073/pnas.2015096118 33608481 PMC7958437

[ece39926-bib-0085] Turesson, G. (1922). The genotypical response of the plant species to the habitat. Hereditas, 3, 211–350. 10.1111/j.1601-5223.1922.tb02734.x

[ece39926-bib-0086] Volis, S. , Yakubov, B. , Shulgina, I. , Ward, D. , & Mendlinger, S. (2005). Distinguishing adaptive from nonadaptive genetic differentiation: Comparison of *Q* _ST_ and *F* _ST_ at two spatial scales. Heredity, 95, 466–475. 10.1038/sj.hdy.6800745 16189543

[ece39926-bib-0087] Waldmann, P. , & Andersson, S. (1998). Comparison of quantitative genetic variation and allozyme diversity within and between populations of *Scabiosa canescens* and *S. columbaria* . Heredity, 81, 79–86. 10.1046/j.1365-2540.1998.00379.x

[ece39926-bib-0088] Whitlock, M. C. (2008). Evolutionary inference from *Q* _ST_ . Molecular Ecology, 17, 1885–1896. 10.1111/j.1365-294X.2008.03712.x 18363667

[ece39926-bib-0089] Wright, S. (1951). The genetical structure of populations. Annals of Eugenics, 1, 323–354. 10.1111/j.1469-1809.1949.tb02451.x 24540312

[ece39926-bib-0090] Ye, Q. , Tang, F. , Wei, N. , & Yao, X. (2014). Molecular and quantitative trait variation within and among small fragmented populations of the endangered plant species *Psilopeganum sinense* . Annals of Botany, 113, 79–86. 10.1093/aob/mct255 24265350 PMC3864726

[ece39926-bib-0091] Yu, Q. , Ellen, E. D. , Wade, M. J. , & Delph, L. F. (2011). Genetic differences among populations in sexual dimorphism: Evidence for selection on males in a dioecious plant. Journal of Evolutionary Biology, 24, 1120–1127. 10.1111/j.1420-9101.2011.02245.x 21401772 PMC3118645

